# Alterations in DNA Methylation in Orofacial Clefts

**DOI:** 10.3390/ijms232112727

**Published:** 2022-10-22

**Authors:** Chirakan Charoenvicha, Wimon Sirimaharaj, Krit Khwanngern, Nipon Chattipakorn, Siriporn C. Chattipakorn

**Affiliations:** 1 Plastic and Reconstructive Surgery Unit, Department of Surgery, Faculty of Medicine, Chiang Mai University, Chiang Mai 50200, Thailand; 2 Clinical Surgical Research Center, Chiang Mai University, Chiang Mai 50200, Thailand; 3 Cardiac Electrophysiology Research and Training Center, Faculty of Medicine, Chiang Mai University, Chiang Mai 50200, Thailand; 4 Center of Excellence in Cardiac Electrophysiology Research, Chiang Mai University, Chiang Mai 50200, Thailand; 5 Neurophysiology Unit, Cardiac Electrophysiology Research and Training Center, Faculty of Medicine, Chiang Mai University, Chiang Mai 50200, Thailand; 6 Department of Oral Biology and Diagnostic Sciences, Faculty of Dentistry, Chiang Mai University, Chiang Mai 50200, Thailand

**Keywords:** cleft lip and palate, orofacial clefts, congenital craniofacial anomalies, DNA methylation, epigenetics

## Abstract

Orofacial clefts are among the most common craniofacial anomalies with multifactorial etiologies, including genetics and environments. DNA methylation, one of the most acknowledged mechanisms of epigenetics, is involved in the development of orofacial clefts. DNA methylation has been examined in patients with non-syndromic cleft lip with cleft palate (nsCL/P) from multiple specimens, including blood, saliva, lip, and palate, as well as experimental studies in mice. The results can be reported in two different trends: hypomethylation and hypermethylation. Both hypomethylation and hypermethylation can potentially increase the risk of nsCL/P depending on the types of specimens and the specific regions on each gene and chromosome. This is the most up-to-date review, intending to summarize evidence of the alterations of DNA methylation in association with the occurrence of orofacial clefts. To make things straightforward to understand, we have systematically categorized the data into four main groups: human blood, human tissues, animal models, and the factors associated with DNA methylation. With this review, we are moving closer to the core of DNA methylation associated with nsCL/P development; we hope this is the initial step to find a genetic tool for early detection and prevention of the occurrence of nsCL/P.

## 1. Introduction

Orofacial clefts are one of the most common birth defects. The incidence of orofacial clefts accounts for up to half of all craniofacial anomalies [1]. Orofacial clefts affect an average of 1 in 700 live births, globally [2]. Orofacial clefts can be categorized into two main types: cleft lip (CL) and cleft palate (CP). Cleft lip is a disruption of the lip tissues, which varies in severity depending on upward extension into the nasal floor. Cleft palate is a disruption of the palatal tissues or the secondary palate, including the hard and/or soft palate [3]. Cleft lip can occur either in association with or without cleft palate. Thus, orofacial clefts can be classified as (1) cleft lip with cleft palate (CL/P); (2) cleft lip only (CLO); and (3) cleft palate only (CPO). The consequences of CL/P affect multiple aspects of the life of a patient, frequently requiring lifelong multidisciplinary healthcare by plastic surgeons, orthodontists, speech pathologists, psychologists, nurses, and other healthcare professionals. This long-term care not only impacts the health/psychological status of patients and their families, but also the overall social and financial burden of the public healthcare system [4]. The best way to approach CL/P treatment is to prevent the occurrence of defects. Therefore, understanding the pathogenesis behind cleft lip/palate will be the best way to prevent or lessen the defects of orofacial clefts.

CL/Ps are also classified as either syndromic or non-syndromic CL/Ps [5]. Syndromic CL/Ps occur in association with other structural abnormalities. Approximately 70% of CL/Ps occur in the absence of other abnormalities and are known as “non-syndromic CL/P (nsCL/P)”. Syndromic CL/Ps are inherited as Mendelian traits [6]; thus, the exact corresponding genetic etiologies of syndromic CL/Ps are known [7]. However, the pathogenesis of nsCL/P is currently elusive. The possible etiology of nsCL/P is multifactorial, varying with the complexity of gene–environment interactions [8,9,10]. Genetic factors of nsCL/P include gene polymorphism and advanced maternal age. The environmental factors include maternal smoking and all-trans retinoic acid exposure. However, the exact true relationships and mechanistic insights between these risk factors and nsCL/P are still unclear.

In this genetic era, one of the possible relationships is “DNA methylation”. DNA methylation is characterized by the addition of a methyl group to cytosine (C) onto the C5 position in DNA, which is a well-known epigenetic modification in mammal cells [11,12,13]. Consequently, the interruption of the folate and methionine metabolized cycles can affect DNA methylation. Alterations in DNA methylation, including both upregulation (or “hypermethylation”) and downregulation (or “hypomethylation”), are associated with craniofacial malformations such as orofacial clefts [14]. The association of DNA methylation with orofacial clefts was first demonstrated in rodents in 1994 [15]. In humans, maternal folic acid deficiency can lead to the deletion of one carbon in the folate metabolized cycle, resulting in hypomethylation [16]. Maternal obesity can lead to demethylation, which increases the risk of orofacial clefts [17]. A recent meta-analysis also reported that differential methylation in mothers who used tobacco resulted in orofacial clefts in their offspring [16].

ORFs arise from a disruption of midface embryologic development during pregnancy. The upper lip is formed in the sixth week of the fetus. Subsequently, the palate develops in the seventh week of the fetus. The different types of clefts with various degrees of severity are suspected to result from different embryologic defects according to the distinct timing of developmental interruptions, and differences in DNA methylation. Even though focusing on DNA methylation in human embryologic development is an initial step, much research is performed in an animal model to avoid the ethical issue of human embryologic study, especially regarding negative harmful factors such as smoking.

Therefore, the present review aims to discuss and summarize the current information on DNA methylation in orofacial clefts through gathering the studies of the embryologic development from animal models to human blood (both cleft patients and their mothers) and human-affected tissues from clinical studies. In addition, genetic and environmental factors which induced DNA methylation in orofacial clefts are included, as well as any controversial findings.

The database search was performed in the PubMed database by using the following keywords: cleft lip and palate, orofacial cleft, DNA methylation, blood, lip and palatal tissue, factors of cleft lip and palate. The relevant articles that were published in English from 1994 to 2021 were retrieved.

## 2. Alterations in DNA Methylation Found in Blood Samples of Patients with anOrofacial Cleft

DNA methylation has unique characteristics, including cell specificity (different cell types with differential methylation), reversible pathways (different times of collection showing different levels of methylation), and fluctuations in response to environmental factors. All of these characteristics result in difficulty identifying one certain tissue biomarker at each specific time point of collection [12]. Thus, one of the most suitable samples to represent overall DNA methylation in nsCL/P is blood [18]. In blood samples of nsCL/P, hypomethylated genes were found in three studies, including (1) Alvizi et al. [18]: MYC (chromosome 8), FAT1 (chromosome 4), Chr1 (chromosome 1), WHSC1 (chromosome 4); (2) Howe et al. [19]: VAX1 and NTN1; and (3) Xu et al. [20]: BICC1 (chromosome 10). Hypermethylation in blood of nsCL/P was also observed. Li et al. [21] reported hypermethylation of LINE-1 and IRF-6, together with LOC146880 from Howe et al. [19] and CLASRP from Xu et al. [20]. Li et al. [21] collected data from the Chinese Defect Network to identify methylation in blood lymphocytes. LINE-1 (long interspersed nucleotide element-1) has been widely accepted as a genomic biomarker and shows a strong correlation with levels of global DNA methylation in congenital anomalies [22]. LINE-1 has been previously reported as showing hypomethylation in cases of neural tube defect [20,21,22,23,24], but it was described as significant hypermethylation in the nsCL/P population. In contrast, Li et al. [21] postulated that the differences in methylated regions of blood cells and different embryonic origins of the body resulted in different patterns of methylation. LINE-1 hypermethylation specifically increased risks of the cleft lip only (CLO), as shown by odd ratio (OR) of CLO: 12.07, OR of CL/P: 6.89, and OR of CLP: 4.83. In addition, IRF-6 (which plays a role in promoting epithelial–mesenchymal transition in lip and palatal fusion) was found to be hypermethylated, and hypermethylated IRF-6 increased the risks of cleft lip and palate (CLP) rather than other types of orofacial clefts, as shown by OR of CLP: 6.0, OR of CL/P: 4.67, and OR of CLO: 3.6 [21]. Hypermethylation of CLASRP also showed a strong correlation with cleft palate only [20].

The mechanism to explain how DNA methylation was correlated with the occurrence of nsCL/P, is still unclear. Howe et al. reported that hypomethylation of VAX1 and NTN1 as well as hypermethylation of LOC146880 led to nsCL/P through the mediation pathway. The mediation pathway starts from a genetic variant called the “single-nucleotide polymorphism”, which causes aberrant DNA methylation, resulting in nsCL/P [19]. These findings are supported by Alvizi et al., who reported that DNA methylation is “a second hit” which occurs following the first hit via gene mutation. Alvizi et al. also found a correlation between different levels of methylation and differences in penetrated signs of nsCL/P. The symptomatic nsCL/P subjects with penetrated signs had higher levels of methylation than those of the subjects who were carriers with non-penetrated signs. Moreover, both symptomatic nsCL/P subjects and the carriers had an incidence of methylation higher than that of the normal population [18].

Each type of cleft has a difference in levels of methylation. Xu et al. reported hypomethylation of gene BICC1 on chromosome 10, which showed a correlation with cleft lip. Conversely, hypermethylation of CLASRP on chromosome 9 was associated with cleft palate [20]. Sharp et al. measured the level of DNA methylation in whole blood of the UK population, at genes TBX1 (chromosome 22), COL11A2 (chromosome 6), HOXA2 (chromosome 7), CRB2 (chromosome 9), PDGFRA (chromosome 4), and CRISPLD2 (chromosome 16) [17]. The authors found a higher level of methylation in the occurrence of cleft palate only (CPO) than cleft lip only (CLO). However, genes SMOC1 (chromosome 14), PVRL1 (chromosome 11), and CCL2 (chromosome 17) had a lower level of methylation in the cleft palate only, when compared with those of cleft lip only [25]. The differences in the methylation between these two types of clefts could be due to the differences in the embryologic origins of both organs. In general, the upper lip is differentiated from medial and lateral nasal prominence between the 4th and 5th week, whereas the palate is differentiated from the palatal shelves between the 5th and 7th week of gestational development.

The differences in methylation between subtypes can be used for early diagnosis in-utero, prior to ultrasonography. The difference in methylation may be useful for detecting an occult lesion, such as a “submucous cleft palate”. This lesion is a type of cleft palate in which the palatal mucosa is intact, but there is a separation of the underlying levator veli palatini muscle and notching of the hard palate. Due to these obscure defects, a submucous cleft palate is one of the most missed diagnoses and cannot be detected until children present with speaking difficulties due to velopharyngeal insufficiency. The late detection of submucous cleft palate is almost too late for surgical correction, and the outcome of surgery is not always successful. Therefore, the identification of higher levels of blood methylation in cleft palate-related genes could be an early biomarker for prenatal nsCL/P diagnosis, which would help with detecting more cases. All of these findings are summarized in Table 1.

## 3. Alterations in DNA Methylation Found in Lip, Palate, and Saliva of Patients with an Orofacial Cleft

Specimens from affected tissue of orofacial clefts, including the lip and palate, demonstrated a trend of changes in methylation associated with blood methylation [25]. Alvizi et al. reported that blood DNA hypomethylation at MYC, FAT1, Chr1, and WHSC1 contributed to cleft lip, so the authors focused on those methylations of lip tissue methylation in their study. The authors collected 18 lip tissues and paired them with 18 blood samples from the same individuals to find a methylation correlation between blood and lip tissue using linear regression analysis. The results showed a highly positive correlation and significant similarity in methylation between lip and blood samples. These findings suggest that subjects with nsCL/Ps had extensive hypomethylation in lip and blood [18]. These results are supported by Sharp et al., who collected whole blood samples from 150 nsCL/Ps (50 CLO, 50 CPO, 50 CL/P); lip tissues from 50 CLOs, 43 lip tissues from CL/Ps; and palatal tissues from 50 CPOs [25]. The results showed a correlation of the methylation between blood and tissues. These correlations were highly significant in the blood and lip tissue of cleft lip patients. Congruently, the methylation of blood and palatal tissue in cleft palate patients showed a higher correlation than that of blood and lip tissue. These findings suggest the different origins of embryologic development of the lip and palatal tissue, resulting in different levels of methylation [25].

Khan et al. emphasized the occurrence of changes in methylation of the population with cleft lip by performing a study known as the PENTACLEFT project, which focused on LINE-1 methylation, a potentially surrogate gene for the measurement of global DNA methylation [26]. Khan et al. found differences in the incidence of methylation between medial side and lateral side of the lip tissue from the same patient with cleft lip. Lip tissue on the medial side of the cleft lip tended to have a higher methylation level than the lateral side (1.87%). This can be explained by embryologic origin since the lateral side of the cleft lip is differentiated from the maxillary process at the 4th week of gestational age and the medial side develops from the medial nasal process at the 5th week. Khan et al. also performed a thorough search for the factor that precipitated LINE-1 aberrant methylation in patients with cleft lip [27]. They confirmed that the medial side of lip methylation (75.13%) was higher than the lateral side (72.18%). They also showed that the variants of methyltetrahydrofolate reductase (MTHFR) gene, particularly at C (not T) allele, caused hypermethylation in the medial side of cleft lip, rather than the lateral side. MTHFR gene produces MTHFR, which is an enzyme responsible for the catalysis of homocysteine to methionine [28]. Hypofunction of the MTHFR gene leads to lower SAM and consequently to hypomethylation [29,30,31]. All of these findings suggest that the polymorphism of MTHFR c.677C > T can increase the risk of nsCL/P.

Age of patients is another factor that strongly affects levels of methylation [32,33]. Sharp et al. also mentioned “Age-related methylation”, in which the level of methylation can change over time [25]. Differences in times of collection of lip samples (from cheiloplasty during lip repair at 3–6 months after birth) and palatal samples (from palatoplasty during palatal repair at 6–18 months), as well as a synergistic effect between times of collection and differences in the origin of tissue samples, resulted in differences in levels of methylation. At the time of lip correction, CLO and CL/P patients were always younger than CPO patients (palatal correction) [25]. Greater alterations in the level of methylation occurred in older patients. Therefore, the level of methylation at the time of birth, but not at the time of lip repair or palatal repair, could represent true embryologic methylation in orofacial clefts.

The most recent study by Young et al. examined six monozygotic twin pairs discordant for nsCL/P [34]. This study directly compared methylation levels between an unaffected normal twin and an affected cleft lip and palate twin by using their saliva. Saliva involves minimally invasive collection and represents a suitable sample for DNA methylation [35] as it reflects the embryologic origin of lip and palatal tissue from neural crest cells [32,36]. Results have revealed the role of extragenic factors, especially differential methylation in the same direction of MAFB and ZEB2. MAFB participates in palatal development, while ZEB2 plays a role in neural crest cell migration and facial development. Young et al. also performed a pathway analysis of the expression of cleft lip and palate through the Hippo signaling pathway [34]. Hippo is one of the key signaling pathways of craniofacial development. All of these findings are shown in Table 2.

## 4. Alterations in DNA Methylation in Animals with a Genetically Induced Orofacial Cleft

Animal models have been selected to represent human embryologic studies. Mice are the most suitable models because of similarities in the developmental processes of lip and palate. Lip formation in mice occurs around mid-gestational age starting from embryonic day (E) 11, and is followed by a fusion of facial ectoderm on E12.5 [37]. The palatal shelf fusion at the midline occurs on E14.5 [38]. Among mouse lines, A/ strains, particularly A/WySn, have been widely used as a representative model for CL/P patients because they have a genetic failure in the fusion of facial prominences, in which approximately 20–30% of those mice develop cleft lip and palate [39]. CL/P in the A/WySn strain is a consequence of the combination of two genes: (1) clf1 on chromosome 11 and (2) clf2 on chromosome 13 [40]. The clf1 gene in A/ strains is associated with intracisternal A particle (IAP) endogenous retroviral transposition of the Wnt9b gene mutation, suggesting that Wnt insufficiency could lead to abnormal growth and fusion of facial prominences [40]. However, the exact underlying mechanism of clf2 is still unknown.

Plamondon et al. investigated the mechanism of clf2 on nsCL/P and the relationship between methylation alterations and cleft subtypes [41]. The genotype of clf1 and clf2 includes the A and B alleles. For the occurrence of nsCL/P, homozygous A clf1 (clf1^AA^) and heterozygous clf2 (clf2^AB^) were observed. The occurrence was found to be increased to a full frequency in cases of homozygous A clf1 (clf1^AA^) combined with homozygous A clf2 (clf2^AA^). By comparing the normal mice (C57BL/6J) with A/WySn mice, Plamondon et al. found that clf2 was the determinant factor for clf1 or Wnt9b methylation level. The methylation level was also different between genotypes. Clf1^BB^ had higher methylation than clf2^AB^, and the lowest methylation was found in the clf1^AA^ genotype. Interestingly, mice with clf1^AA^ genotype showed an increased occurrence of nsCL/P. There were differences not only in the level of methylation between the different genotypes, but also in the phenotypes of the lip. The level of methylation in normal mice with normal lips was between 30–60%. However, the level of methylation in the nsCL/P group (0–20%) was significantly decreased when compared to that of normal mice [40]. The authors also reported similar patterns of methylation levels from the lip compared to normal lip (50%), the unilateral cleft lip phenotype (10–40%), and the bilateral cleft lip phenotype (0%, or unmethylation). These findings suggest that the lowering of methylation levels could lead to an incidence of the more severe phenotype of cleft lip.

The other mechanism of clf2 on orofacial clefts in the A/WySn mice is via the deficiency of Wnt9b. Juriloff et al. hypothesized that aberrant methylation in clf2 caused Wnt9b gene silencing and interfered in the Wnt9b transcription process, leading to defects of lip and palate or cleft phenomena [42]. This study was conducted through a comparison of levels of Wnt9b transcription at each developmental process between normal mice and A/WySn mice. At E10 (nasal pit invagination), the transcription level of Wnt9b in A/WySn mice was lower than that of normal mice by 1.6 fold. At a later stage of the developmental periods (E11: periods of medial and lateral nasal prominence expansion), the level of Wnt9b transcription in A/WySn mice was also lower than that of normal mice by 2.2 fold. In addition, levels of Wnt9b transcription in the process of primary palate formation on E12 of A/WySn mice were lower than that of normal mice by 1.5 fold.

Green et al. focused on the relationship between levels of Wnt9b expression and lip phenotype [37]. In A/WySn mice, 25% of all embryos had lower levels of Wnt9b methylation (10–20%). The differences in levels of methylation were significantly associated with the variation of facial shape of orofacial clefts. Furthermore, more changes in methylation were associated with an increase in the severity of orofacial clefts. All of these findings are summarized in Table 3.

## 5. Alterations in DNA Methylation in Animals with a Genetically Induced Orofacial Cleft

Changes in DNA methylation can occur in response to external influences, such as environmental factors. Abnormality in DNA methylation during pregnancy can also pass to the developing fetus [12].

Maternal cigarette smoking is one of the most important teratogens and is well documented as a risk of nsCL/P [18]. Mukhopadhyay et al. extracted first branchial arch-derived cells (1-BA cells) of ICR mice (normal mice) following the application of cigarette smoke extract (CSE) [43]. The first branchial arch in week 4 of human gestation is a precursor of the maxillary process, which finally develops into the upper lip and palate during weeks 7 to 10. DNA methylation is primarily regulated by DNA methyltransferases (DNMTs), which are the key enzymes for catalyzation in cooperation with methyl CpG binding (MBD) proteins, acting as modulators to maintain normal methylation. Mukhopadhyay et al. showed a significant decrease in global DNA methylation of 1-BA cells with CSE incubation, as indicated by up to 13% decreased levels of DNA methylation with a high dose of CSE (80 mcg/mL) [43]. CSE administration also decreased the expression of DNMTs and MBD proteins and genes. These reductions in cigarette smoke extract occur through the proteomic degradation pathway; thus, pretreatment with a proteasomal inhibitor reversed the CSE effects.

Retinoic acid (RA), another chemical teratogen, is a group of vitamin A metabolites [44]. Retinoid-like agents are used as active ingredients in common medications, including acne treatment and some cosmetic skin creams. Thus, exposure to RA is easy and potentially frequent in daily life. Exogenous retinoids (including high doses of vitamin A) can disrupt embryologic development (including lip formation and palatogenesis) and alter DNA methylation, resulting in cleft lip and palate [45]. Shu et al. focused on the effects of all-trans retinoic acid (ATRA), a metabolite form of vitamin A, on the induction of cleft palate in mice [46]. They showed hypermethylation of the HDAC4 and SMAD3 genes in normal mice after oral gavage with ATRA. HDAC4 plays a role in the development of skeletal muscles. SMAD3 is essential in epithelial proliferation and regulation of the Wnt signaling pathway. Shu and colleagues also found that hypomethylation of the MID1 gene induced microtubule depolymerization. Hypermethylation of both HDAC4 and SMAD3 genes with hypomethylation of the MID1 gene had an impact on the cis-acting element and the region of non-coding DNA. These defects impaired gene transcription and caused silencing in transcription and inhibiting protein synthesis, finally resulting in the disruption of palatogenesis and increased risk of cleft palate.

The effect of ATRA on palatogenesis had been studied by Shu et al. [38]. They investigated detailed morphology of specific genes (Fgf16 and Tbx22) on the palatogenesis. Their results showed that normal mice with ATRA had complete palatal shelf separation at E14.5, with hypermethylation of both Fgf16 and Tbx22. Fgf16 (fibroblast growth factor 16) plays a role in the regulation of palatal rugae development. ATRA induced Fgf16 hypermethylation and caused gene suppression as well as apoptosis of mouse embryonic palatal mesenchymal cells. All of the events following ATRA treatment resulted in palatal defects. The expression of Tbx22 gene specifically causes palatal shelf mesenchymal tissue proliferation and palatal shelf elevation. The hypermethylation of Tbx22 was found to cause a decrease in gene expression and impaired palatogenesis with consequences of cleft palate. Shu et al. suggested that ATRA induced hypermethylation of Fgf16 and Tbx22, leading to a decrease in the expression of Fgf16 and Tbx22, and increased the risk of cleft palate [38].

Recent evidence supports the finding that maternal folic acid deficiency is associated with the incidence of not only neural tube defects, but also orofacial clefts [47,48]. Folic acid is a requisite agent, as a carbon donor substance, which is regulated through the normal process of methylation. Levels of folic acid in pregnancies with in utero exposure are also directly affected by DNA methylation level of their offspring [49,50]. Gonseth et al. explored the preventative effects of maternal folic acid supplements on aberrant DNA methylation in the newborn, and the incidence of nsCL/P, by using archived newborn bloodspots who were born between 1988 and 1997 (before mandatory dietary folate fortification in the US) [51]. The results showed widespread hypomethylation of up to 63% in the nsCL/P group [51]. The most hypomethylated genes were ZNF77 and EDH2 on chromosome 19. In addition, the most changed sites of DNA methylation are on “Metastable epialleles”, which are vulnerable to maternal nutrition. In addition to the widespread hypomethylation, there are two hypermethylated sites on MIR140 and Wnt9b. A previous study in zebrafish reported that loss of MIR140 function resulted in disruption of neural crest cell accumulation and palatal formation, causing craniofacial abnormalities.

In humans, a decrease in the expression of MIR140 in the first trimester during pregnancy with a history of passive smoking was associated with an increased risk of nsCL/P [52]. Wnt9b was previously mentioned by Juriloff et al. [42] in 2014 and Green et al. [37] in 2019, who reported that it is essential in craniofacial development in mice, especially in the A/WySn model. Gonseth et al. suggested that MIR140 and Wnt9b are associated with nsCL/P since the absence of these variations was observed in neural tube defects [50]. All of these findings are reported in Table 4.

**Figure 1:**
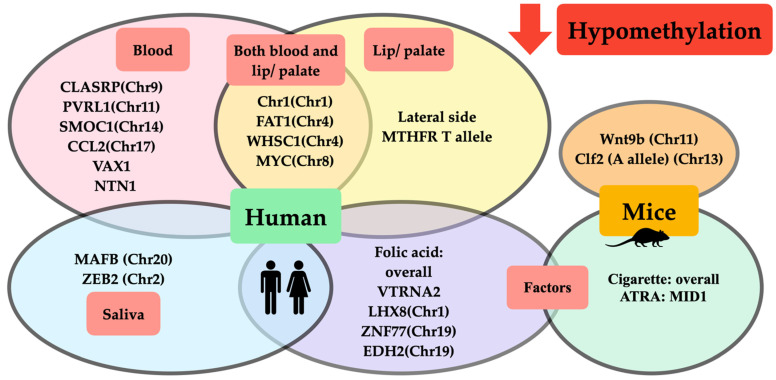
Schematic representation of DNA hypomethylation in orofacial clefts from animal and clinical studies. Hypomethylation from clinical studies demonstrated that (1) hypomethylated genes and chromosomes in human blood (pink circle) were CLASRP(Chr9), PVRL1 (Chr11), SMOC1(Chr11), CCL2(Chr17), VAX1, and NTN1; (2) hypomethylated genes and chromosomes in lip/ palatal tissue (yellow circle) were lateral side of lip tissue, MTHFR T allele; (3) hypomethylated genes and chromosomes found either in blood or lip/palatal tissue (intersection area between pink and yellow circle) were Chr1(Chr1), FAT1(Chr4), WHSC1(Chr4), and MYC(Chr8); (4) hypomethylated genes and chromosomes in saliva (blue circle) were MAFB(Chr20) and ZEB2(Chr2); and (5) factors affecting DNA hypomethylation in clinical studies (purple circle) included folic acid affecting hypomethylation of overall methylation of VTRNA2, LHX8(Chr1), ZNF77(Chr19), and EDH2(Chr19). Hypomethylation from animal studies demonstrated that (1) hypomethylated genes and chromosomes in mice (orange circle) were Wnt9b(Chr11) and Clf2(A allele)(Chr13); and (2) factors affecting DNA hypomethylation in studies on mice (green circle) included cigarettes affecting hypomethylation of overall DNA methylation and ATRA affecting hypomethylation of MID1.

**Figure 2:**
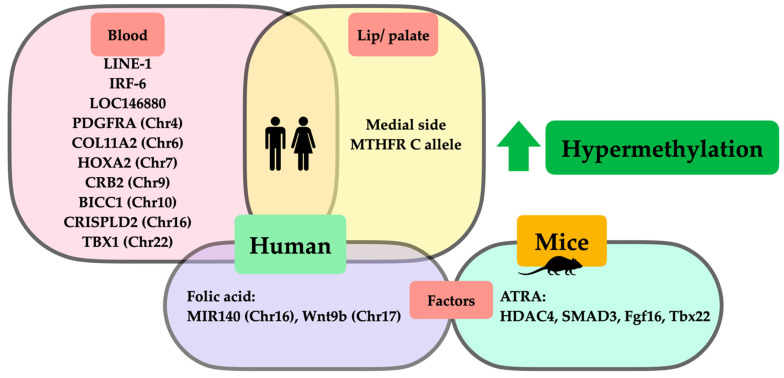
Schematic representation of DNA hypermethylation in orofacial clefts from animal and clinical studies. Hypermethylation from clinical studies demonstrated that (1) hypermethylated genes and chromosomes in human blood (pink square) were LINE-1, IRF-6, LOC146880, PDGFRA(Chr4), COL11A2(Chr6), HOXA2(Chr7), CRB2(Chr9), BICC1(Chr10), CRISPLD2(Chr16), and TBX1(Chr22); (2) hypermethylated genes and chromosomes in lip/palatal tissues (yellow square) were medial side of lip tissues, MTHFR C allele; and (3) factors affecting DNA hypermethylation in clinical studies (purple square) were folic acid affecting hypermethylation of MIR140(Chr16) and Wnt9b(Chr17). Regarding hypermethylation in animal studies, factors affecting DNA hypermethylation in studies on mice (green circle) included ATRA affecting hypermethylation of HDAC4, SMAD3, Fgf16, and Tbx22.

Regarding all evidence that we have previously mentioned, we have summarized those hypomethylated and hypermethylated genes associated with orofacial clefts from both animal and clinical studies in Figure 1 and Figure 2, respectively.

## 6. Limitations and Strengths

In this review, some limitations need to be considered. Firstly, the reviewed DNA methylation data are scattered between humans and animals, tissue-specific samples, and variations through time. Thus, a conclusion pointing to one main trend or single gene may ignore the varied nature of the data and lead to oversimplification. Secondly, the environmental risks in different intensities and periods of time are inevitable confounding factors. This results in different methylations and ultimately different phenotypes of nsCL/P.

Despite these limitations, a strength of this review is the highlighting of methylation changes, in an effort to summarize two main trends: hyper- and hypomethylation. The data span from the embryologic to postnatal period, with surrogated animal models combining the effects of both genes and maternal environments with different types of tissue samples (blood, lip, palate). Given the reproducible methylation changes in orofacial clefts (such as overall methylation), both blood and affected lip and palatal tissues from orofacial cleft patients and their mothers should be further investigated, since DNA methylation in mother blood could be a predictive biomarker for orofacial clefts in the baby. We hope the findings of this review will clarify and inform the initial steps for further study.

## 7. Conclusions

Orofacial clefts, especially nsCL/P, have been a result of multifactorial genetic and environment interactions, in which the combination of genetics and environment can be called “epigenetics”. Epigenetics plays an important role in the evolution of all kinds of living things. DNA methylation, known as the most relevant epigenetic modification in mammals, has the effects of silencing gene activity and disrupting protein synthesis. DNA methylation is acknowledged as being one of the factors involved in the development of orofacial clefts. Studies have demonstrated several alterations in DNA methylation found in orofacial clefts, from which we can summarize that there are two types of methylation associated with orofacial clefts: hypomethylation and hypermethylation. Each type of aberrant DNA methylation depends on the type of tissue and time of tissue collection. Understanding DNA methylation in orofacial clefts will contribute to the development of new genetic tools for early detection, the accumulation of prognostic information, and eventually, the prevention of the occurrence of orofacial clefts.

## Figures and Tables

**Table 1:** Alterations in DNA methylation found in blood samples of patients with an orofacial cleft.

Samples (Population)	Number of Cases: Gender (Controls: Gender)	Major Findings in Methylation					Interpretation	Ref
		Gene (Chromosome)	CpG	DMR	Methylation Level (%)	Risk of Orofacial Cleft		
Whole blood (Brazil) Whole blood (UK)	67 CL/P: M = 37, F = 30 (59: M = 28, F = 31) 171 CL/P: M = 107, F = 64 (177: M = 100, F = 77)	*(Chr8)* *(Chr4)* *(Chr1)* *(Chr4)*	cg00611675 cg00405769 cg15897635 cg03150409	MYC FAT1 Chr1 WHSC1	↓ ↓ ↓ ↓	NA	Hypomethylation at MYC, FAT1, Chr1, WHSC1 contributed to CLP.	Alvizi et al., 2017 [18]
Leukocytes (UK)	Total 150: 50 CLO: M = 31, F = 19 50 CPO: M = 27, F = 23 50 CL/P: M = 42, F = 8 (-)	*TBX1(Chr22)* *COL11A2 (Chr6)* *HOXA2 (Chr7)* *CRB2 (Chr9)* *PDGFRA (Chr4)* *CRISPLD2 (Chr16)* *SMOC1 (Chr14)* *PVRL1 (Chr11)* *CCL2 (Chr17)*	6 15 7 2 2 2 5 2 6	19750918–19752870 33132086–33132728 27143046–27143807 126130901–126131310 55090812–55091179 84870066–84870204 70316898–70317240 119630144–119630]3 32582128–32582829	↑ CPO vs. CLO ↑ CPO vs. CLO ↑ CPO vs. CLO, CL/P ↑ CPO vs. CLO ↑ CPO vs. CLO ↑ CPO vs. CLO ↓ CPO vs. CL/P ↓ CPO vs. CLO ↓ CPO vs. CLO	NA	Methylation level associated with orofacial cleft subtype, mostly CPO, was hypermethylated to a greater extent than CLO (dependent on regions of methylation).	Sharp et al., 2017 [25]
Leukocytes (China)	Total 37: M = 28, F = 9 20 CL/P: - 17 CLO: - (60: M = 40, F = 20)	*LINE-1 (-)* *IRF-6 (-)*	L1_11.12 I6_34.35	NA NA	Total (↑ 1.78%) >CL/P (↑ 1.77%) >CLO (↑ 1.75%) >control CL/P (↑ 3.42%) >Total (↑ 2.99%) >CLO (↑ 2.48%) >control	OR: ↑ CLO 12.07 OR: ↑ Total 6.89 OR: ↑ CL/P 4.83 OR: ↑ CL/P 6.00 OR: ↑ Total 4.67 OR: ↑ CLO 3.60	Hypermethylation of LINE-1 and IRF-6 increased CL/P risk.	Li et al., 2019 [21]
Whole blood (UK)	Central European: 399 CL/P: - (1318: -) European ICC duos/trios: 816 CL/P: - (1454: -)	*VAX1 (-)* *NTN1 (-)* *LOC146880 (-)*	cg11398452 cg16197528 cg02598441	NA	↓ ↓ ↑	Mediation causal effect: SNP → DNA methylation → CL/P	Genetic variants caused hypomethylation of VAX1 and NTN1 and hypermethylation of LOC146880; both induced CL/P.	Howe et al., 2019 [19]
Whole blood (Norway)	92 CLO: M = 51, F = 41; 84 CPO: M = 37, F = 47; 132 CL/P: M = 95, F = 37 (436: M = 238, F = 198)	*BICC1 (Chr10)* *(CLO + CL/P)* *CLASRP (Chr9)* *(CPO + CL/P)*	cg09696939 cg26985354	26 31	↓ (average 0.02) ↑ (average 0.89)	NA NA	BICC1 hypermethylation was associated with cleft lip subtype; CLASRP hypomethylation was associated with cleft palate subtype.	Xu et al., 2019 [20]

Abbreviations: CLP—cleft lip with cleft palate; CLO—left lip only; CPO—cleft palate only; DMR—differential methylated region; CpG—cytosine–guanine methylation area; SNP—single-nucleotide polymorphism; ICC—International Consortium to Identify Genes and Interactions Controlling Oral Clefts; OR—odds ratio; F—female; M—male; NA—not available; ↓—decreased; ↑—increased.

**Table 2:** Alterations in DNA methylation found in lip, palatal tissue, and saliva of patients with an orofacial cleft.

Samples (Population)	Number of Cases (Controls)	Major Findings in Methylation				Interpretation	Ref
		Gene (Chromosome)	CpG (DMR)	Methylation Level (%)	Correlation with Blood Methylation		
Lip (UK)	18 CL/P lip (18 CL/P blood from same individuals)	NA	NA	NA	Highly positive	A correlation was shown between methylation and lip and blood samples	Alvizi et al., 2017 [18]
Lip (UK) Palate (UK)	48 CLO (43 CL/P) 7 CPO (43 CL/P)	*LOC154882 (Chr7)* *PARK2* *(Chr6)* *OR2L13* *(Chr1)* *KIAA0415 (Chr7)* *VAMP3* *(Chr1)* NA	- (158789723–158790116) - (161796785–161796855) - (248100183–248100615) - (4832112–4832536) - (7842159–7842407) NA	NA NA NA CLO 4% > CL/P NA NA	84% positive 73% positive	Blood methylation levels showed a strong correlation with tissue-specific cleft subtype: - Between blood and lip in CLO - Between blood and palate in CPO	Sharp et al., 2017 [25]
Lip (China)	Total 23 CL 13 CL/P 10 (-)	*LINE-1 (-)*	NA	Medial > lateral (↑1.87%)	NA	Two separated embryologic origins and timing of development of CL caused LINE-1 methylation profile differences in which the medial side was hypermethylated more than the lateral side.	Khan et al., 2018 [26]
Lip (China)	45 CL/P: 23 left, 6 right, 7 bilateral (-)	*LINE-1 (-)* *MTHFR c* *.677C > T genotype* *-CC* *-CT* *-TT*	NA	Medial > lateral (↑4.3%) Medial > lateral (↑3.14%) ↔	NA	LINE-1 methylation in the medial side of CL was hypermethylated to a greater extent than the lateral side, which was possibly influenced by *MTHFR* c.677C > T (C allele).	Khan et al., 2019 [27]
Saliva (US)	6 affected CLP twin (6 unaffected normal twin)	*MAFB (Chr20)* *ZEB2 (Chr2)*	NA	↓	NA	Comparison of differential methylation between monozygotic twin pairs discordant for NSCLP found hypomethylation in MAFB and ZEB2.	Young et al., 2021 [34]

Abbreviations: CL/P—cleft lip with cleft palate; CL—cleft lip; DMR—differential methylated region; CpG—cytosine–guanine methylation area; NA—not available; ↓—decreased; ↑—increased.

**Table 3:** Alterations in DNA methylation in animals with a genetically induced orofacial cleft.

Animal Model Cases (Control)	Number of Cases (Control)	Age of Embryo (Organ Specificity)	Major Findings in Methylation			Interpretation	Ref
			Gene (Chromosome)	Transcription Level	Methylation Level (Mean %)		
A/WySn mice, C57BL/6J mice (-)	1146 (-)	E14 (lip formation)	Clf2 (Chr13) Genotype BB, AB, AA	NA	BB > AB > AA A/WySn lip phenotype - ↑ Normal lip (30–60%) -↓ CL/P (0–20%)	- Clf2 gene polymorphism, especially in A allele, induced hypomethylation and an increased risk of CL/P in A/WySn mice strain. - Severity of lip phenotype varied throughout methylation levels of Clf2 (lower methylation directly correlated with increased severity of lip phenotypes).	Plamondon et al., 2011 [41]
A/WySn mice (C57BL/6J mice as normal strain)	Transcription level - 13/ (6) Methylation level - 12/ (10)	E10 (nasal pit invagination) E11 (medial and lateral prominence expansion) E12 (primary palate formation)	Wnt9b (Chr11)	A/WySn < C57BL/6J 1.6 fold A/WySn < C57BL/6J 2.2 fold A/WySn < C57BL/6J 1.5 fold	↓ Overall A/WySn lip phenotype (compared with C57BL/6J) - ↑ Normal lip (50%) - ↓ Unilateral CL (10–40%) - ↓↓ Bilateral CL (0%)	- A/WySn mice had low Wnt9b transcription level and increased risk of CL. - Hypomethylation of Wnt9b was correlated with the severity of CL phenotypes (lower methylation was associated with more severe phenotypes).	Juriloff et al., 2014 [42]
A/WySn mice (-)	50 (-)	E11 (medial nasal process)	Wnt9b (Chr11)	NA	↓ (0–20%)	Hypomethylation at Wnt9b in A/WySn mice nonlinearly affected facial shape variation in CLP (lower methylation was associated with increased facial shape variation).	Green et al., 2019 [37]

Abbreviations: CL/P—cleft lip with cleft palate; CL—cleft lip; E—embryologic age; NA—not available; ↓—decreased; ↑—increased.

**Table 4:** Alterations in DNA methylation regarding environmental risk factors that induced orofacial cleft.

Species /Organ Specificity (Age) /Population	Risk Factors /Exposure duration /Vehicle	Number of Cases (Control)	Major Findings in Methylation				Interpretation	Ref
			Gene (Chromosome)	CpG (DMR)	Gene Expression Level (Fold Change)	Methylation Level (% Change)		
ICR mice (normal strain) /1st branchial arch cultured cell (E10.5) /-	Cigarette smoke extract (CSE) 20 mcg/mL 40 mcg/mL 80 mcg/mL /24 h /Phosphate-buffered saline Proteasome inhibitor (MG-132) 1.5 mcM /3 h prior to 24 h of CSE /Dimethyl sulfoxide	NA NA	Overall Overall Overall DNMT-1 (-) DNMT-3A (-) DNMR-3B (-) MeCP-2 (-) MBD-2 (-) MBD-3 (-) DNMT-1 (-) DNMT-3A (-) MeCP-2 (-) MBD-3 (-)	NA NA	NA NA NA ↓ 2.21 ↓ 1.85 ↓ 1.80 ↓ 2.43 ↓ 1.50 ↓ 1.52 ↑ ↑ ↑ ↑	↔ ↓ 1.8 % ↓ 13% NA NA NA NA NA NA NA NA NA NA	Cigarette smoke extract decreased overall gene expression level of DNA methyltransferase and methyl-CPG-binding domain protein, leading to global DNA hypomethylation in a dose-dependent manner, which induced occurrence of CLP. Pretreatment with proteasomal inhibitor can reverse global DNA methyltransferase and methyl-CPG-binding domain protein degradation from cigarette smoke extract via inhibition of the proteomic degradation pathway.	Mukhopadhyay et al., 2015 [43]
C57BL/6J mice (normal strain) /palatal shelves (E14.5) /-	All-trans retinoic acid 70 mg/kg /4 days oral gavage /corn oil	3 (3)	HDAC4 (-) SMAD3 (-) MID1 (-)	92051600–92053400 63658601–63660200 169978000–169980801	NA	↑ ↑ ↓	All-trans retinoic acid caused hypermethylation of both HDAC4 and SMAD3 and hypomethylation of MID1, which disrupted palatogenesis, resulting in an increased risk of CP.	Shu et al., 2018 [46]
C57BL/6J mice (normal strain) /palatal shelves (E14.5) /-	All-trans retinoic acid 70 mg/kg /oral gavage /corn oil	3 (3)	Fgf16 (ChrX) Tbx22 (-)	105725515–105764278 CCGG exon sequences	↓ ↓	↑ ↑	All-trans retinoic acid caused reciprocal relation (hypermethylation but decreased gene expression) of Fgf16 and Tbx22, which caused developmental failure of the palate, leading to CP.	Shu et al., 2019 [38]
Human /newborn archived bloodspot /USA	Without folic acid (prior to mandatory folate dietary) /- /-	94 CL/P: M = 57, F = 37 (88: M = 51, F = 37)	NA ZNF77 (Chr19) EDH2 (Chr19) VTRNA2-1 (-) LHX8 (Chr1) MIR140 (Chr16) WNT9B (Chr17)	63.5% of overall CpG cg19689947 cg02718229 NA NA NA NA	NA	↓ ↓↓ ↓↓ ↓ ↓ ↑ ↑	Periconceptional folic acid deficiency induced widespread hypomethylation, except hypermethylation of MIR140 and WNT9B, which increased the risk of CL/P.	Gonseth et al., 2019 [51]

Abbreviations: CL/P—cleft lip with cleft palate; CLO—cleft lip only; CPO—cleft palate only; DMR—differential methylated region; CpG—cytosine–guanine methylation area; F—female; M—male; E—embryologic age; DNMT—DNA methyltransferase; MBD—methyl-CPG-binding domain protein; NA—not available. ↓—decreased; ↑—increased.

## Abbreviations

Chr—chromosome; ATRA—all-trans retinoic acid; MTHFR—methyltetrahydrofolate reductase; LINE-1—long interspersed nucleotide element-1; Fgf16—fibroblast growth factor1.

## References

[1] Stanier P., Moore G.E. (2004). Genetics of cleft lip and palate: Syndromic genes contribute to the incidence of non-syndromic clefts. Hum. Mol. Genet..

[2] Mossey P.A., Modell B. (2012). Epidemiology of oral clefts 2012: An international perspective. Front. Oral. Biol..

[3] Dixon M.J., Marazita M.L., Beaty T.H., Murray J.C. (2011). Cleft lip and palate: Understanding genetic and environmental influences. Nat. Rev. Genet..

[4] Lace B., Vasiljeva I., Dundure I., Barkane B., Akota I., Krumina A. (2006). Mutation analysis of the MSX1 gene exons and intron in patients with nonsyndromic cleft lip and palate. Stomatologija.

[5] Kohli S.S., Kohli V.S. (2012). A comprehensive review of the genetic basis of cleft lip and palate. J. Oral Maxillofac. Pathol..

[6] Spritz R.A. (2001). The genetics and epigenetics of orofacial clefts. Curr. Opin. Pediatr..

[7] Mossey P.A., Little J., Munger R.G., Dixon M.J., Shaw W.C. (2009). Cleft lip and palate. Lancet.

[8] Garland M.A., Sun B., Zhang S., Reynolds K., Ji Y., Zhou C.J. (2020). Role of epigenetics and miRNAs in orofacial clefts. Birth Defects Res..

[9] Cantone I., Fisher A.G. (2013). Epigenetic programming and reprogramming during development. Nat. Struct. Mol. Biol..

[10] Sharp G.C., Stergiakouli E., Sandy J., Relton C. (2018). Epigenetics and Orofacial Clefts: A Brief Introduction. Cleft Palate Craniofac. J..

[11] Seelan R.S., Pisano M., Greene R.M. (2019). Nucleic acid methylation and orofacial morphogenesis. Birth Defects Res..

[12] Lei H., Oh S.P., Okano M., Jüttermann R., Goss K.A., Jaenisch R., Li E. (1996). De novo DNA cytosine methyltransferase activities in mouse embryonic stem cells. Development.

[13] Jones P.A. (2012). Functions of DNA methylation: Islands, start sites, gene bodies and beyond. Nat. Rev. Genet..

[14] Greene R.M., Pisano M.M. (2010). Palate morphogenesis: Current understanding and future directions. Birth Defects Res. Part C Embryo Today Rev..

[15] Rogers J.M., Francis B.M., Sulik K.K., Alles A.J., Massaro E.J., Zucker R.M., Elstein K.H., Rosen M.B., Chernoff N. (1994). Cell death and cell cycle perturbation in the developmental toxicity of the demethylating agent, 5-aza-2’-deoxycytidine. Teratology.

[16] Joubert B.R., Felix J.F., Yousefi P., Bakulski K.M., Just A.C., Breton C., Reese S.E., Markunas C.A., Richmond R.C., Xu C.J. (2016). DNA Methylation in Newborns and Maternal Smoking in Pregnancy: Genome-wide Consortium Meta-analysis. Am. J. Hum. Genet..

[17] Sharp G.C., Lawlor D.A., Richmond R.C., Fraser A., Simpkin A., Suderman M., Shihab H.A., Lyttleton O., McArdle W., Ring S.M. (2015). Maternal pre-pregnancy BMI and gestational weight gain, offspring DNA methylation and later offspring adiposity: Findings from the Avon Longitudinal Study of Parents and Children. Int. J. Epidemiol..

[18] Alvizi L., Ke X., Brito L.A., Seselgyte R., Moore G.E., Stanier P., Passos-Bueno M.R. (2017). Differential methylation is associated with non-syndromic cleft lip and palate and contributes to penetrance effects. Sci. Rep..

[19] Howe L.J., Richardson T.G., Arathimos R., Alvizi L., Passos-Bueno M.R., Stanier P., Nohr E., Ludwig K.U., Mangold E., Knapp M. (2019). Evidence for DNA methylation mediating genetic liability to non-syndromic cleft lip/palate. Epigenomics.

[20] Xu Z., Lie R.T., Wilcox A.J., Saugstad O.D., Taylor J.A. (2019). A comparison of DNA methylation in newborn blood samples from infants with and without orofacial clefts. Clin. Epigenetics.

[21] Li Y., Deng Y., Deng C., Xie L., Yu L., Liu L., Yuan Y., Liu H., Dai L. (2019). Association of long interspersed nucleotide element-1 and interferon regulatory factor 6 methylation changes with nonsyndromic cleft lip with or without cleft palate. Oral Dis..

[22] Xu M., Wu X., Li Y., Yang X., Hu J., Zheng M., Tian J. (2014). CITED2 mutation and methylation in children with congenital heart disease. J. Biomed. Sci..

[23] Chen X., Guo J., Lei Y., Zou J., Lu X., Bao Y., Wu L., Wu J., Zheng X., Shen Y. (2010). Global DNA hypomethylation is associated with NTD-affected pregnancy: A case-control study. Birth Defects Res. Part A Clin. Mol. Teratol..

[24] Wang L., Wang F., Guan J., Le J., Wu L., Zou J., Zhao H., Pei L., Zheng X., Zhang T. (2010). Relation between hypomethylation of long interspersed nucleotide elements and risk of neural tube defects. Am. J. Clin. Nutr..

[25] Sharp G.C., Ho K., Davies A., Stergiakouli E., Humphries K., McArdle W., Sandy J., Davey Smith G., Lewis S.J., Relton C.L. (2017). Distinct DNA methylation profiles in subtypes of orofacial cleft. Clin. Epigenetics.

[26] Khan M.F.J., Little J., Mossey P.A., Steegers-Theunissen R.P., Autelitano L., Lombardo I., Andreasi R.B., Rubini M. (2018). Evaluating LINE-1 methylation in cleft lip tissues and its association with early pregnancy exposures. Epigenomics.

[27] Khan M.F.J., Little J., Aleotti V., Mossey P.A., Steegers-Theunissen R.P.M., Autelitano L., Meazzini M.C., Ravaei A., Rubini M. (2019). LINE-1 methylation in cleft lip tissues: Influence of infant MTHFR c.677C>T genotype. Oral Dis..

[28] Mills J.L., Kirke P.N., Molloy A.M., Burke H., Conley M.R., Lee Y.J., Mayne P.D., Weir D.G., Scott J.M. (1999). Methylenetetrahydrofolate reductase thermolabile variant and oral clefts. Am. J. Med. Genet..

[29] Prescott N.J., Winter R.M., Malcolm S. (2002). Maternal MTHFR genotype contributes to the risk of non-syndromic cleft lip and palate. J. Med. Genet..

[30] Behunova J., Klimcakova L., Podracka L. (2011). Urinary tract anomalies associated with MTHFR gene polymorphism C677T in girls. Kidney Blood Press. Res..

[31] Blanton S.H., Patel S., Hecht J.T., Mulliken J.B. (2002). MTHFR is not a risk factor in the development of isolated nonsyndromic cleft lip and palate. Am. J. Med. Genet..

[32] Simpkin A.J., Suderman M., Gaunt T.R., Lyttleton O., McArdle W.L., Ring S.M., Tilling K., Davey Smith G., Relton C.L. (2015). Longitudinal analysis of DNA methylation associated with birth weight and gestational age. Hum. Mol. Genet..

[33] Acevedo N., Reinius L.E., Vitezic M., Fortino V., Söderhäll C., Honkanen H., Veijola R., Simell O., Toppari J., Ilonen J. (2015). Age-associated DNA methylation changes in immune genes, histone modifiers and chromatin remodeling factors within 5 years after birth in human blood leukocytes. Clin. Epigenetics.

[34] Young J.I., Slifer S., Hecht J.T., Blanton S.H. (2021). DNA Methylation Variation Is Identified in Monozygotic Twins Discordant for Non-syndromic Cleft Lip and Palate. Front. Cell. Dev. Biol..

[35] Smith A.K., Kilaru V., Klengel T., Mercer K.B., Bradley B., Conneely K.N., Ressler K.J., Binder E.B. (2015). DNA extracted from saliva for methylation studies of psychiatric traits: Evidence tissue specificity and relatedness to brain. Am. J. Med. Genet. Part B Neuropsychiatr. Genet..

[36] Patel V.N., Hoffman M.P. (2014). Salivary gland development: A template for regeneration. Semin. Cell Dev. Biol..

[37] Green R.M., Leach C.L., Diewert V.M., Aponte J.D., Schmidt E.J., Cheverud J.M., Roseman C.C., Young N.M., Marcucio R.S., Hallgrimsson B. (2019). Nonlinear gene expression-phenotype relationships contribute to variation and clefting in the A/WySn mouse. Dev. Dyn..

[38] Shu X., Dong Z., Cheng L., Shu S. (2019). DNA hypermethylation of Fgf16 and Tbx22 associated with cleft palate during palatal fusion. J. Appl. Oral Sci..

[39] Juriloff D.M., Harris M.J. (2008). Mouse genetic models of cleft lip with or without cleft palate. Birth Defects Res. Part A Clin. Mol. Teratol..

[40] Juriloff D.M., Harris M.J., Dewell S.L., Brown C.J., Mager D.L., Gagnier L., Mah D.G. (2005). Investigations of the genomic region that contains the clf1 mutation, a causal gene in multifactorial cleft lip and palate in mice. Birth Defects Res. Part A Clin. Mol. Teratol..

[41] Plamondon J.A., Harris M.J., Mager D.L., Gagnier L., Juriloff D.M. (2011). The clf2 gene has an epigenetic role in the multifactorial etiology of cleft lip and palate in the A/WySn mouse strain. Birth Defects Res. Part A Clin. Mol. Teratol..

[42] Juriloff D.M., Harris M.J., Mager D.L., Gagnier L. (2014). Epigenetic mechanism causes Wnt9b deficiency and nonsyndromic cleft lip and palate in the A/WySn mouse strain. Birth Defects Res. Part A Clin. Mol. Teratol..

[43] Mukhopadhyay P., Greene R.M., Pisano M.M. (2015). Cigarette smoke induces proteasomal-mediated degradation of DNA methyltransferases and methyl CpG-/CpG domain-binding proteins in embryonic orofacial cells. Reprod. Toxicol..

[44] Liu S., Higashihori N., Yahiro K., Moriyama K. (2015). Retinoic acid inhibits histone methyltransferase Whsc1 during palatogenesis. Biochem. Biophys. Res. Commun..

[45] Kuriyama M., Udagawa A., Yoshimoto S., Ichinose M., Sato K., Yamazaki K., Matsuno Y., Shiota K., Mori C. (2008). DNA methylation changes during cleft palate formation induced by retinoic acid in mice. Cleft Palate Craniofac. J..

[46] Shu X., Shu S., Zhai Y., Zhu L., Ouyang Z. (2018). Genome-Wide DNA Methylation Profile of Gene cis-Acting Element Methylations in All-trans Retinoic Acid-Induced Mouse Cleft Palate. DNA Cell Biol..

[47] Erickson R.P. (2010). Genes, environment, and orofacial clefting: N-acetyltransferase and folic acid. J. Craniofac. Surg..

[48] Liu H.Y., Liu S.M., Zhang Y.Z. (2020). Maternal Folic Acid Supplementation Mediates Offspring Health via DNA Methylation. Reprod. Sci..

[49] Richmond R.C., Sharp G.C., Herbert G., Atkinson C., Taylor C., Bhattacharya S., Campbell D., Hall M., Kazmi N., Gaunt T. (2018). The long-term impact of folic acid in pregnancy on offspring DNA methylation: Follow-up of the Aberdeen Folic Acid Supplementation Trial (AFAST). Int. J. Epidemiol..

[50] López-Gordillo Y., Maldonado E., Nogales L., Del Río A., Barrio M.C., Murillo J., Martínez-Sanz E., Paradas-Lara I., Alonso M.I., Partearroyo T. (2019). Maternal folic acid supplementation reduces the severity of cleft palate in Tgf-β(3) null mutant mice. Pediatr. Res..

[51] Gonseth S., Shaw G.M., Roy R., Segal M.R., Asrani K., Rine J., Wiemels J., Marini N.J. (2019). Epigenomic profiling of newborns with isolated orofacial clefts reveals widespread DNA methylation changes and implicates metastable epiallele regions in disease risk. Epigenetics.

[52] Bliek B.J., Steegers-Theunissen R.P., Blok L.J., Santegoets L.A., Lindemans J., Oostra B.A., Steegers E.A., de Klein A. (2008). Genome-wide pathway analysis of folate-responsive genes to unravel the pathogenesis of orofacial clefting in man. Birth Defects Res. Part A Clin. Mol. Teratol..

